# A Rare Association of Pituitary Macroadenoma With Nasopharyngeal Angiofibroma: A Case Report

**DOI:** 10.7759/cureus.45565

**Published:** 2023-09-19

**Authors:** Jasleen Kaur, Prasad T Deshmukh, Shraddha Jain, Chandra Veer Singh, Sagar S Gaurkar

**Affiliations:** 1 Department of Otorhinolaryngology, Jawaharlal Nehru Medical College, Datta Meghe Institute of Higher Education and Research, Wardha, IND

**Keywords:** nasal mass, acromegaly, pituitary macroadenoma, angiofibroma, epistaxis

## Abstract

Pituitary macroadenoma and angiofibroma are two distinct and diverse types of tumors that can develop in different anatomical locations and clinical characteristics and are not typically related to each other in terms of their hormonal or developmental aspects. This case describes an adult male with pituitary macroadenoma with nasal angiofibroma. A 35-year-old male was diagnosed with pituitary macroadenoma and incidentally found to have juvenile nasopharyngeal angiofibroma (NPA). The patient underwent a diagnostic workup, including imaging studies and hormonal assays, which confirmed the concomitant presence of both tumors. The patient underwent successful endoscopic surgical excision of the NPA and transnasal transsphenoidal endoscopic pituitary macroadenoma excision as a two-stage operation. The patient was followed up postoperatively and had no evidence of tumor recurrence or hormonal imbalances. The importance of complete and comprehensive diagnostic workup and multidisciplinary management in achieving successful and optimum treatment outcomes for coexisting NPA and pituitary macroadenoma in an adult patient is highlighted in the present report.

## Introduction

Nonfunctioning type pituitary adenomas are the second most frequent form of tumor of pituitary origin [1]. These neoplasms account for approximately 15% of primary intracranial tumors. There occurs secretion of hormones due to proliferation of pituitary cells which leads to a spectrum of endocrine symptoms [2]. These tumors can cause a range of symptoms, stemming from their size, hormone production disruption, and pressure on surrounding structures. Understanding the common symptoms associated with pituitary macroadenomas is vital for early detection and effective management of these growths [3]. It is crucial to diagnose these neoplasms and further refer to centres where relevant expertise is available to optimize the short as well as long-term consequences of these patients [4].

Nasopharyngeal angiofibroma is an extremely vascular lesion and may have one or more than that arterial vascular pedicles [5]. It is usually mentioned as juvenile nasopharyngeal angiofibroma (JNA), angiofibromatous or fibromatous hamartoma of the nasal cavity or juvenile angiofibroma (JAF) [6]. The internal maxillary artery provides the primary arterial supply followed by the ascending pharyngeal artery, both being branches of the external carotid artery, accessory meningeal, middle meningeal and facial artery branches are the additional accessory arteries supplying the growth. Lesions that are larger in size may reduce multiple feeding arteries comprising even bilateral involvement. The conscription of branches of the internal carotid artery with the vidian artery being the most frequent, and to a lesser extent the ophthalmic artery are also described by the researchers [5]. The etiopathogenesis of the tumor is not entirely understood but, as a majority of the patients with nasopharyngeal angiofibroma are males, hence high androgen receptor (AR) expression can be elucidated, suggesting that JNA is androgen dependent. Furthermore, vascular and hamartoma malformation theories are also suggested as once the JNA histologic origin involves endothelial cells or fibroblasts [6]. The co-occurrence of nasopharyngeal angiofibroma and pituitary macroadenoma is rare and can present unique diagnostic and treatment challenges.

## Case presentation

**Figure 1:**
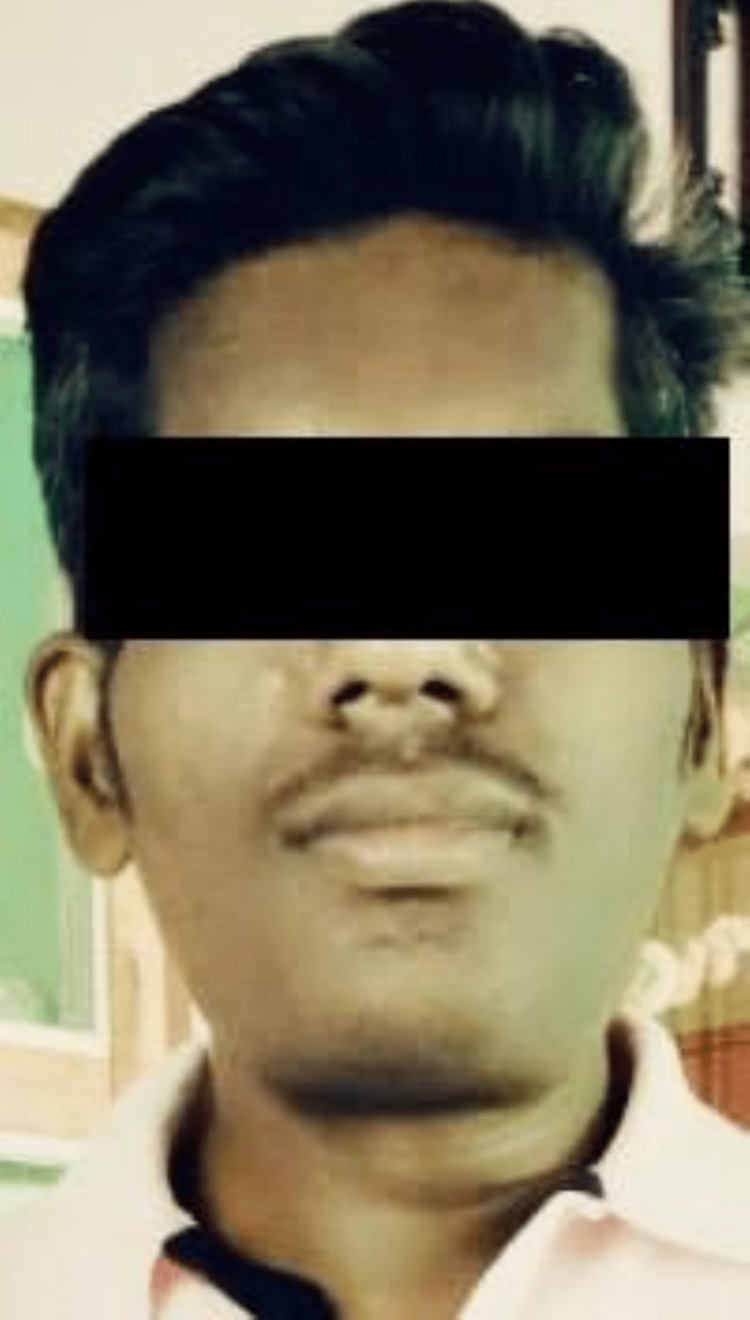
A two-year-old photograph with normal facial features

**Figure 2:**
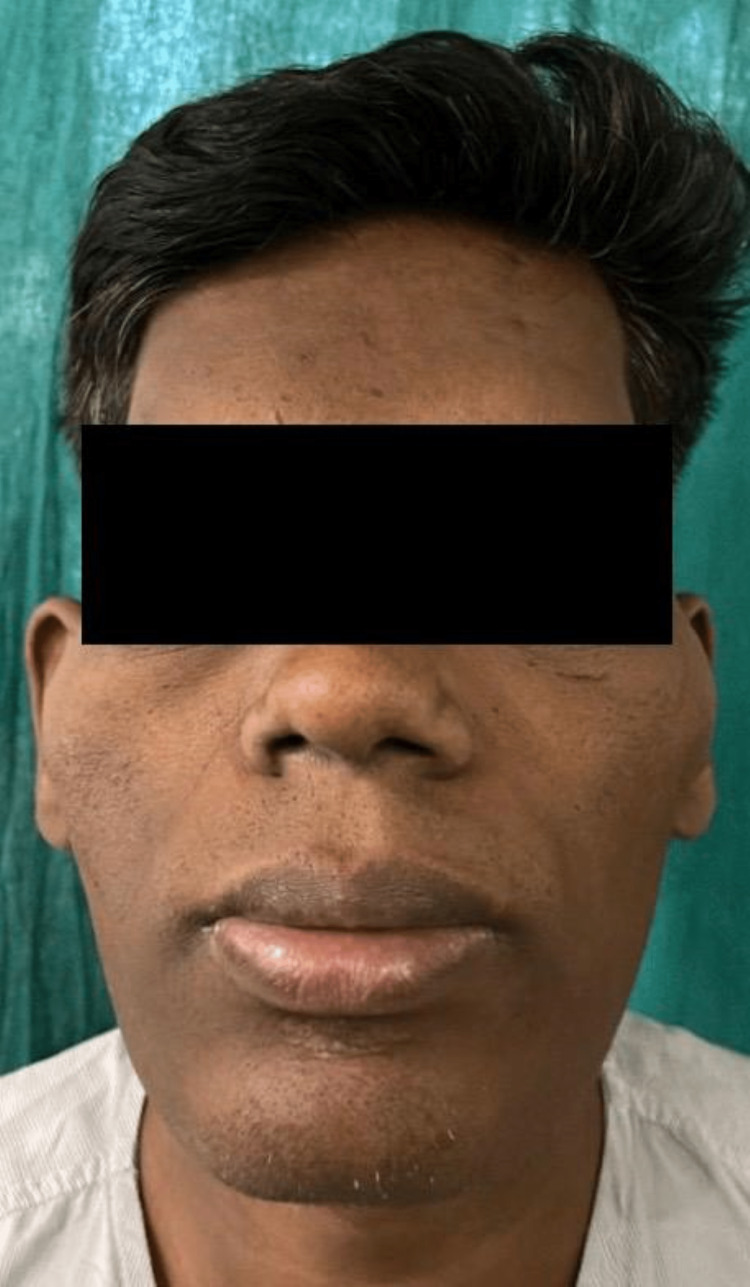
A recent photograph showing coarse facial features, a prominent forehead, pronounced jaw, enlarged lips and nose, malar prominence, and thickened skin

A 35-year-old male presented to the ENT OPD with a complaint of recurrent right nasal bleeding for the past two years. The patient reported experiencing painless episodes of epistaxis lasting for 15 to 20 minutes each time, resulting in blood loss of 30 to 40 ml. He also complains of mucopurulent nasal discharge for three months occasionally. Epistaxis was profuse and was managed by anterior nasal packing and blood transfusion. The patient had been experiencing headaches, vision problems, and reduced visual field with diminution of vision at the periphery for several months. The physical examination of the patient revealed prominent supraorbital ridges, a broad nose, mandibular prognathism, thickened lips and thickened skin folds on the scalp, which are consistent with acromegalic facial features. In the backdrop of these older photographs, facial features were conspicuously different, when compared with recent photographs. Figure 1 shows a two-year-old photograph, while Figure 2 depicts a recent picture obtained in our hospital showing coarse facial features, a prominent forehead, pronounced jaw, enlarged lips and nose, malar prominence, and thickened skin. 

**Figure 3:**
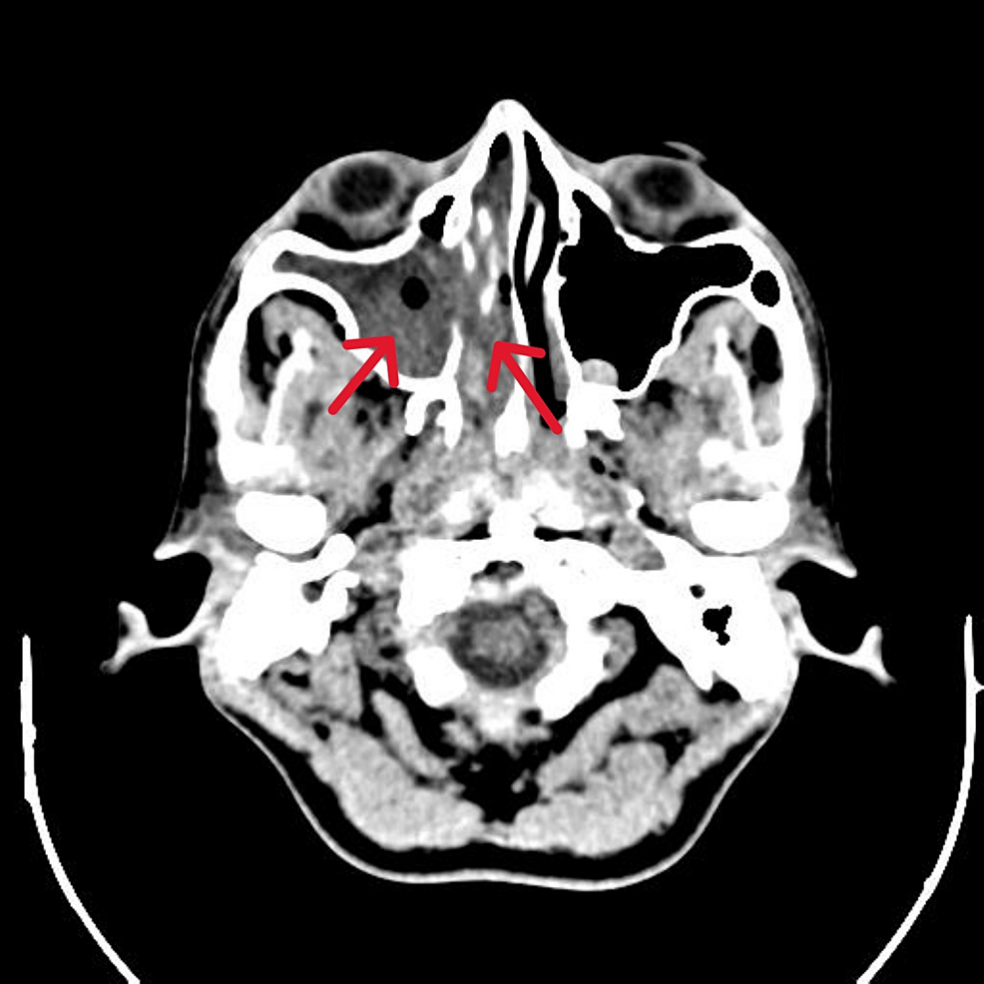
CT paranasal sinus (PNS) axial view shows evidence of paranasal sinus enlargement with remodelling and destruction of sinus walls and nasal turbinates, suggestive of nasopharyngeal angiofibroma (grade 2 A) (red arrow)

**Figure 4:**
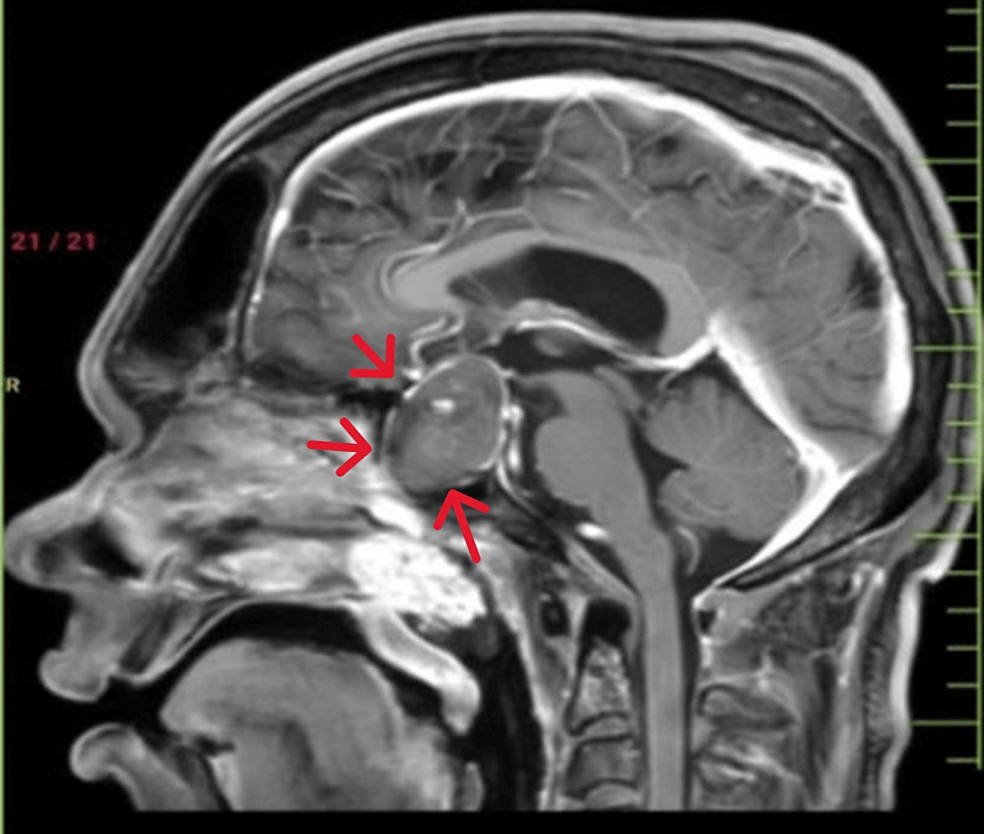
Contrast-enhanced MRI (CE-MRI) Brain showing heterogenous enhancement with altered signal intensity was observed in the sellar and suprasellar region, approximately measuring 4x3.7×3cm pituitary macroadenoma (red arrow)

Magnetic Resonance Imaging (MRI) and Computed Tomography (CT) scans (Figure 3) of the brain and skull base were done which revealed pituitary macro adenoma. MRI Brain (Figure 4) had evidence of heterogeneously increasing, extra-axial altered signal intensity mass lesion observed in the sellar and suprasellar region, approximately measuring 4x3.7×3cm (giving snowman appearance); appearing varied hyperintense on T2-weighted image/fluid-attenuated inversion recovery (T2WI/FLAIR), isointense on T1WI with few areas of hyperintensities foci within and few areas of blooming on susceptibility-weighted imaging (SWI), representing hemorrhage. The lesion was obliterating the optic recess. It was causing a mass effect in the form of optic chiasma superiorly and right carotid vessel laterally. The lesion was causing partial encasement of the left carotid vessel (Knosp grade 2). It was also seen displacing and compressing the third ventricle, causing the widening of the sella and scalloping its wall. Also had evidence of mucosal thickening in the bilateral maxillary (right>left), ethmoid, and right frontal sinus with remodelling and destruction of sinus walls and nasal turbinates, suggestive of nasopharyngeal angiofibroma (grade 2 A).

**Figure 5:**
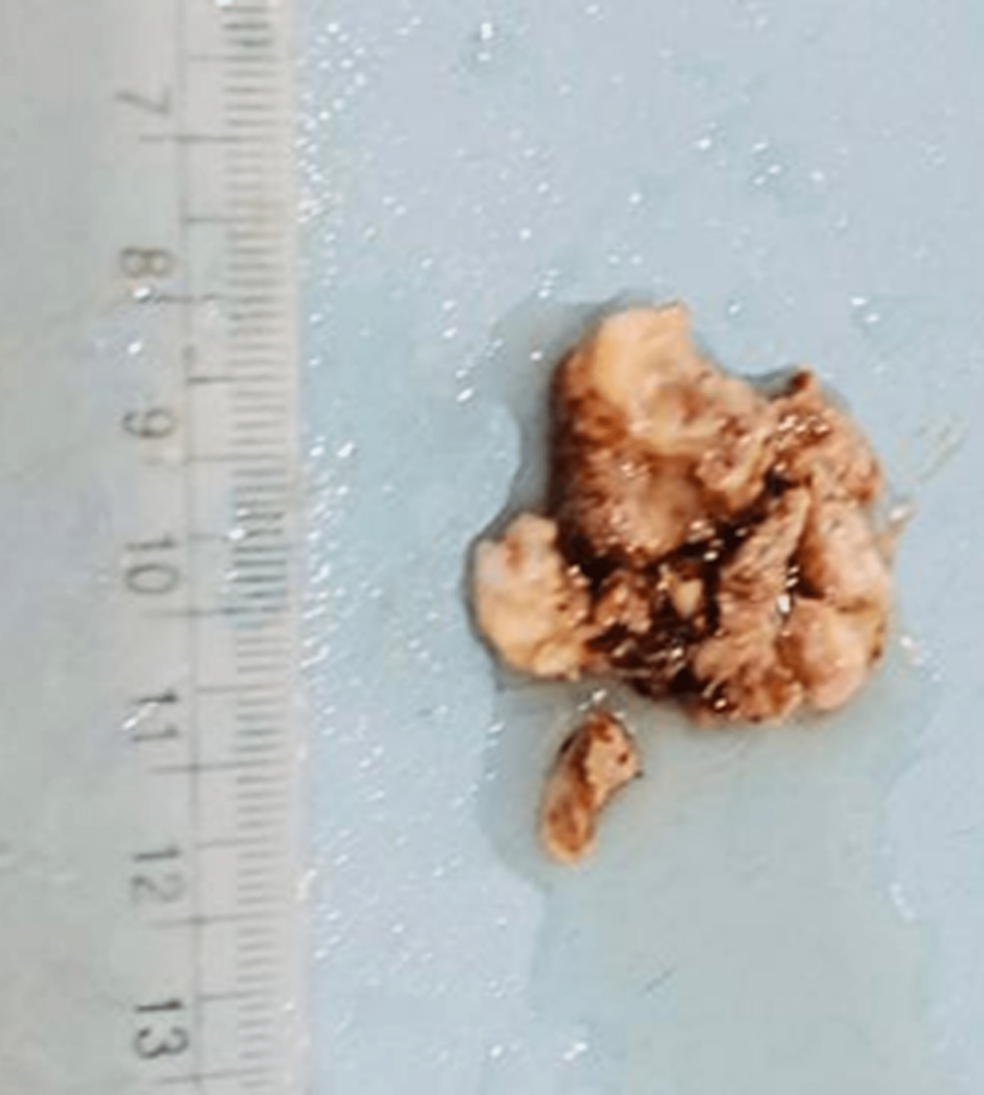
Macroscopic examination revealed a mass of approximately 3.5x4 cm

Functional endoscopic sinus surgery (FESS) was planned for the removal of nasal mass as first-stage surgery. On nasal endoscopy, mucopurulent nasal discharge was visualized arising from the middle meatus. Intraoperatively, a fleshy, vascular mass measuring approximately 2.5x 2 cm seen arising from the right middle turbinate was excised, and a diagnosis of angiofibroma was made. Histopathological examination of the mass revealed angiofibroma. Gross examination revealed a mass of approximately 3.5x4 cm (Figure 5).

**Figure 6:**
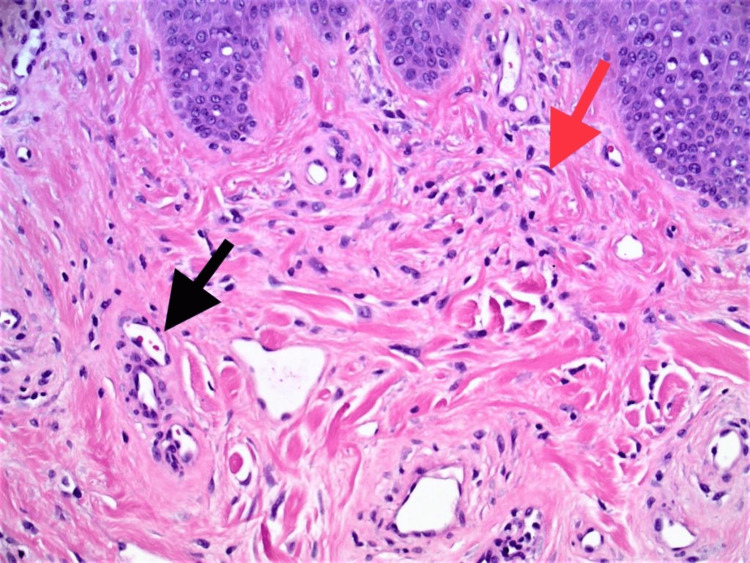
H&E stained section (100 x) reveals myxoid and collagenous alternating areas, with bland spindle cells that are uniform. Spindle cells (red arrow) show eosinophilic cytoplasm. There is evidence of prominent vasculature (black arrow)

On histopathological examination, it was observed that there were polypoid tumoral masses present, which were unencapsulated and composed of stromal connective tissue, covered by a respiratory-type mucosa, and highly vascularized with blood vessels having a multitude of shapes and dimensions ranging from a vascular-slit aspect to an arteriolar aspect; the vascular wall was uneven in thickness, consisting of a layer of endothelial cells and a muscular tunic of varied thickness, and some areas of hyaline and collagen deposits were identified among the muscular cells, but no elastic limits or pericytes were present (Figure 6).

The first stage of the operation involved endoscopic resection of the angiofibroma. The surgery was performed gainfully with the removal of the tumor in its entirety. The patient underwent close monitoring for bleeding and any postoperative distress. Nasal packing was removed after 24 hours. The patient received counseling regarding the necessity of a second-stage surgery to achieve full recovery. The patient had an uneventful recovery and was discharged from the hospital after five days. Symptoms improved after surgery.

Hormonal workup was done, including testosterone (140ng/dl), prolactin (15ng/ml), cortisol (2 microgram/dl); thyroid profile was found to be normal. The second stage of the operation involved transnasal transsphenoidal endoscopic excision of pituitary macroadenoma surgery. This surgery was performed six weeks after the first surgery to allow for proper healing. The tumor size along with its proximity to vital structures made it critical and challenging, nonetheless, the team was able to remove the tumor completely with no major complications. Patient was rigorously monitored in the immediate post-operative phase to assess vital signs, neurological condition, and the presence of any potential complications, including cerebrospinal fluid (CSF) leakage or bleeding. Hormonal analysis yielded normal results, and an MRI was conducted to confirm the absence of any residual mass.

Postoperatively, the patient had a gradual recovery. He was followed up regularly to monitor the postoperative course, and MRI scans showed no recurrence of the tumors.

The successful two-staged operation for the incidental angiofibroma with pituitary macro adenoma highlights the complex situation of twin lesions existing together. The use of advanced imaging techniques, meticulous surgical planning, and careful execution by the surgical team was crucial in achieving a positive outcome for this patient

## Discussion

This case describes an adult male with pituitary macroadenoma and nasal angiofibroma. Pituitary adenomas, with most of them being benign and slow-growing, are the tumors of anterior pituitary. These are categorized based on size and on the basis of size or cell of origin and can be designated as microadenoma, macroadenoma and giant tumors. Microadenoma describes a tumor less than 10 mm, whereas macroadenomas are considered as tumors greater than 10mm [7]. In the present case, gross examination revealed a mass more than 10 mm in size. The large tumors that are bigger than 40 mm are called giant pituitary tumors.

These pituitary adenomas are of two types, functioning and non-functioning. The functioning one causes enlarged secretions of single or multiple hormones of the anterior pituitary depending on their cell type are functional whereas alternative to this, the non-functioning type does not secrete hormones, however there is the possibility that these can compress the adjacent areas of the anterior pituitary leading to hormonal deficiencies. Hence, patients with pituitary adenoma are required to be analysed by a multidisciplinary team that must comprise specialists in endocrinology, ophthalmology and neurosurgery [7].

Adenomas of pituitary are the most frequent source of a mass in the sella with approximately 9% of cases reported with other etiologies for mass lesions in the sellar region. The differential diagnosis for lesions in the sellar and suprasellar area is diverse and consists of neoplastic pathologies such pituitary adenoma, Rathke's cleft cyst, metastases or craniopharyngioma. The other manifestations of these are meningioma, vascular lesions, and inflammatory illnesses like sarcoidosis and autoimmune hypophysitis. Therefore, it is essential to consider this wide range of possibilities while evaluating such cases. Precise preoperative diagnosis is crucial clinically as the preferred treatment for various nonpituitary sellar masses is different from that of a pituitary tumor [8].

Patients with pituitary macroadenomas should undergo a thorough evaluation for hypopituitarism, which involves measuring serum levels of pituitary hormones. The diagnosis of acromegaly is inveterated on a biochemical basis, which includes increased serum insulin-like growth factor 1 as well as lack of suppression of growth hormone after administration of glucose. Pituitary magnetic resonance imaging is recommended in cases with acromegaly to recognise an underlying pituitary adenoma [9]. Facial feature analysis based on photographs of patients (Figures 1, 2) is a useful tool for identifying acromegaly, particularly in patients with subtle symptoms.

The first-line therapy for patients with acromegaly is generally transsphenoidal pituitary surgery. However, patients with macroadenomas are frequently not remitted postoperatively. Medical therapies that comprise somatostatin receptor ligands, pegvisomant and cabergoline can be recommended to patients with relentless disease after surgery. Radiation therapy is mainly a third-line choice and is progressively administered by various stereotactic techniques.

Assessing the para-sellar extension of a macroadenoma is essential in preoperative MRI studies. Para-sellar involvement in pituitary macroadenomas can be difficult to diagnose due to limited clinical features. Direct visualization of dural penetration on MRI can also be challenging. To determine whether an adenoma has infiltrated the cavernous sinus rather than causing lateral bulging, the Knosp criterion and other MRI evaluation standards have been created (Figure 4). These criteria assess certain MRI characteristics, such as venous space encroachment, internal carotid artery encasement, and lateral intercarotid line crossing. A greater probability of cavernous sinus invasion has also been linked to the absence of some venous compartments on an MRI. In order to assess the level of para-sellar involvement in pituitary macroadenomas, MRI assessment is essential [10].

According to Mario-Enrquez and Fletcher's 2012 analysis of 37 instances, juvenile angiofibroma (JA) is a benign fibrovascular neoplasm that makes up 0.5% of all head and neck tumors. The clinical presentation typically involves unilateral epistaxis, nasal obstruction, and an intranasal mass. However, it can also present as a mass in the back, chest wall, abdominal cavity, or pelvic cavity. Epistaxis, or nosebleed, can range from mild to severe and may require various treatments such as nasal packing, vasopressors, antifibrinolytics, and even transfusions [11]. Juvenile nasopharyngeal angiofibromas are staged using cross-sectional imaging to evaluate tumor extent and its invasion into neighboring spaces. The commonly employed staging system divides these tumors into three stages for clinical assessment, In Stage I, tumors are either confined to the nasal cavity/nasopharynx (Ia) or extend into one or more paranasal sinuses (Ib). Stage II includes minimal extension through the sphenopalatine foramen into the pterygopalatine fossa (IIa), filling the pterygopalatine fossa and pushing the posterior wall of the maxillary antrum forward or extending into the orbit via the inferior orbital fissure (IIb), or further extending beyond the pterygopalatine fossa into the infratemporal fossa (IIc). Stage III signifies intracranial extension (Figure 3). This staging system assists clinicians in accurately assessing tumor location and guides treatment planning. JNA is a tumor that primarily occurs in young males and is treated through surgical excision. The surgical approaches for JNA include endoscopic, endoscopic-assisted, or open-surgical techniques. Open surgical techniques involve various procedures such as lateral rhinotomy, trans palatal, trans axillary, midfacial degloving, Le Fort I, Denker, infratemporal, and combinations of these approaches. However, endoscopic resection for JNA has also been investigated and found to be effective in several studies [12]. In the present case, the patient had complaint of recurrent right nasal bleeding for the past two years. The patient reported experiencing painless episodes of epistaxis lasting for 15 to 20 minutes each time, resulting in blood loss of 30 to 40 ml.

Pituitary macroadenomas and angiofibroma are two distinct types of tumors that can occur in the head and neck region, and their co-occurrence is not well established. But, a genetic condition i.e., multiple endocrine neoplasia type 1 (MEN1) is associated with an increased risk of angiofibroma and pituitary tumors [13]. Tumors can form in a variety of endocrine organs, including the pituitary gland and the nasal cavity, when menin function is lost [14]. Another common theory is the vascular system. Angiofibromas are highly vascular tumors, meaning that they contain many blood vessels. Pituitary macroadenomas can also cause the release of hormones that affect the blood vessels in the body, leading to changes in blood flow and vessel growth. Both tumors have been associated with increased blood vessel density and abnormal blood vessel growth which may contribute to their co-development. Additionally, various studies have hypothesised a connection between the expression of specific cytokines and growth factors that are essential for the aetiology and development of angiofibroma and pituitary macroadenomas. Among the cytokines linked to the expansion and development of pituitary macroadenomas and angiofibromas are insulin-like growth factor-2 (IGF2) and transforming growth factor beta-1 (TGFβ-1). In particular, TGFβ-1 has been shown to promote growth by stimulating angiogenesis and inhibiting apoptosis [5], while IGF2 stimulates cell growth and proliferation through the activation of the PI3K/AKT/mTOR pathway [15]. Overall, while the link between these two tumors is not well established, the possibility of common underlying mechanisms suggests that further research may shed some light on twin yet unrelated pathological conditions.

## Conclusions

We presented a case report on co-existence of angiofibroma and pituitary macroadenoma. While both are rare, their occurrence together is the rarest of rare situations. This can prod researchers and healthcare professionals to gain insight into the potential relationship between these tumors and better understanding of the underlying mechanisms that may contribute to their development. It highlights the importance of early diagnosis and appropriate management, along with long-term follow-up among patients with these conditions. It also contributes to comprehending the pathogenesis of tumors of this type of origin which serves as a basis for further research into the relationship between these two conditions, potentially leading to the development of new diagnostic or treatment approaches.
